# Supplementary material for: A custom phenotypic profile for Fanconi anemia: Addressing gaps in existing disease annotations
Source: medRxiv. 2026 Feb 12:2026.02.10.26346018. Preprint. [Version 1] doi: 10.64898/2026.02.10.26346018 (PMC12919158; doi:10.64898/2026.02.10.26346018)
Supplement: Supplement 1 [file media-1.pdf]

Table S1. HPO terms in OMIM, Orphanet, and custom profiles and their corresponding anatomical system.

HPO ID	Term label	OMIM	Orphanet	Custom	Anatomical system
HP:0000010	*Recurrent urinary tract infections*		X		Genitourinary system
HP:0000013	*Hypoplasia of the uterus*			X	Genitourinary system
HP:0000023	*Inguinal hernia*	X			Digestive system
HP:0000027	*Azoospermia*		X	X	Genitourinary system
HP:0000028	*Cryptorchidism*	X	X	X	Genitourinary system
HP:0000029	*Testicular atrophy*			X	Genitourinary system
HP:0000035	*Abnormal testis morphology*		X		Genitourinary system
HP:0000041	*Chordee*			X	Genitourinary system
HP:0000047	*Hypospadias*		X	X	Genitourinary system
HP:0000054	*Micropenis*	X			Genitourinary system
HP:0000062	*Ambiguous genitalia*	X			Genitourinary system
HP:0000072	*Hydroureter*		X	X	Genitourinary system
HP:0000075	*Renal duplication*	X			Genitourinary system
HP:0000076	*Vesicoureteral reflux*	X			Genitourinary system
HP:0000077	*Abnormality of the kidney*			X	Genitourinary system
HP:0000079	*Abnormality of the urinary system*		X		Genitourinary system
HP:0000081	*Duplicated collecting system*	X			Genitourinary system
HP:0000083	*Renal insufficiency*		X		Genitourinary system
HP:0000085	*Horseshoe kidney*	X		X	Genitourinary system
HP:0000086	*Ectopic kidney*	X		X	Genitourinary system
HP:0000089	*Renal hypoplasia*	X		X	Genitourinary system
HP:0000104	*Renal agenesis*	X		X	Genitourinary system
HP:0000107	*Renal cyst*	X			Genitourinary system
HP:0000110	*Renal dysplasia*			X	Genitourinary system
HP:0000122	*Unilateral renal agenesis*	X			Genitourinary system
HP:0000125	*Pelvic kidney*	X			Genitourinary system
HP:0000126	*Hydronephrosis*	X		X	Genitourinary system
HP:0000130	*Abnormality of the uterus*		X		Genitourinary system
HP:0000132	*Menorrhagia*			X	Genitourinary system
HP:0000133	*Gonadal dysgenesis*			X	Genitourinary system
HP:0000135	*Hypogonadism*	X	X		Endocrine system
HP:0000140	*Abnormality of the menstrual cycle*			X	Genitourinary system
HP:0000141	*Amenorrhea*			X	Genitourinary system
HP:0000144	*Decreased fertility*			X	Genitourinary system
HP:0000148	*Vaginal atresia*			X	Genitourinary system
HP:0000151	*Aplasia of the uterus*	X		X	Genitourinary system
HP:0000155	*Oral ulcer*			X	Head or neck
HP:0000175	*Cleft palate*	X	X	X	Head or neck

HP:0000189	*Narrow palate*	X			Head or neck
HP:0000212	*Gingival overgrowth*			X	Head or neck
HP:0000215	*Thick upper lip vermilion*	X			Head or neck
HP:0000217	*Xerostomia*			X	Head or neck
HP:0000218	*High palate*		X	X	Head or neck
HP:0000230	*Gingivitis*			X	Head or neck
HP:0000238	*Hydrocephalus*	X	X	X	Nervous system
HP:0000252	*Microcephaly*	X	X	X	Head or neck
HP:0000268	*Dolichocephaly*		X		Musculoskeletal system
HP:0000280	*Coarse facial features*	X			Head or neck
HP:0000286	*Epicanthus*	X	X	X	Head or neck
HP:0000294	*Low anterior hairline*	X			Head or neck
HP:0000307	*Pointed chin*			X	Head or neck
HP:0000316	*Hypertelorism*	X	X	X	Eye
HP:0000324	*Facial asymmetry*		X		Head or neck
HP:0000325	*Triangular face*	X		X	Head or neck
HP:0000340	*Sloping forehead*		X		Head or neck
HP:0000347	*Micrognathia*	X	X	X	Musculoskeletal system
HP:0000358	*Posteriorly rotated Ears*			X	Ear
HP:0000364	*Hearing abnormality*		X		Ear
HP:0000365	*Hearing impairment*	X	X	X	Ear
HP:0000369	*Low-set ears*	X		X	Ear
HP:0000377	*Abnormal pinna morphology*		X	X	Ear
HP:0000396	*Overfolded helix*	X			Ear
HP:0000402	*Stenosis of the external auditory canal*			X	Ear
HP:0000405	*Conductive hearing impairment*	X			Ear
HP:0000413	*Atresia of the external auditory canal*	X		X	Ear
HP:0000414	*Bulbous nose*	X			Head or neck
HP:0000426	*Prominent nasal bridge*	X			Head or neck
HP:0000430	*Underdeveloped nasal alae*	X			Head or neck
HP:0000431	*Wide nasal bridge*	X			Head or neck
HP:0000437	*Depressed nasal tip*	X			Head or neck
HP:0000452	*Choanal stenosis*	X			Head or neck
HP:0000453	*Choanal atresia*		X		Head or neck
HP:0000463	*Anteverted nares*	X			Head or neck
HP:0000465	*Webbed neck*	X			Integument
HP:0000470	*Short neck*	X			Musculoskeletal system
HP:0000478	*Abnormality of the eye*		X		Eye
HP:0000482	*Microcornea*	X		X	Eye
HP:0000483	*Astigmatism*	X	X		Eye
HP:0000486	*Strabismus*	X	X	X	Eye
HP:0000492	*Abnormal eyelid morphology*		X		Head or neck
HP:0000504	*Abnormality of vision*		X		Eye
HP:0000505	*Visual impairment*		X		Eye
HP:0000506	*Telecanthus*			X	Head or neck

HP:0000508	*Ptosis*		X	X	Head or neck
HP:0000518	*Cataract*		X	X	Eye
HP:0000520	*Proptosis*		X		Head or neck
HP:0000527	*Long eyelashes*	X			Integument
HP:0000543	*Optic disc pallor*	X			Eye
HP:0000545	*Myopia*	X			Eye
HP:0000568	*Microphthalmia*	X	X	X	Eye
HP:0000581	*Blepharophimosis*	X			Head or neck
HP:0000582	*Upslanted palpebral fissure*	X	X		Head or neck
HP:0000598	*Abnormality of the ear*			X	Ear
HP:0000601	*Hypotelorism*	X		X	Eye
HP:0000609	*Optic nerve hypoplasia*	X			Nervous system
HP:0000639	*Nystagmus*		X		Eye
HP:0000689	*Dental malocclusion*	X			Musculoskeletal system
HP:0000691	*Microdontia*			X	Musculoskeletal system
HP:0000692	*Tooth malposition*			X	Musculoskeletal system
HP:0000696	*Delayed eruption of permanent teeth*			X	Musculoskeletal system
HP:0000704	*Periodontitis*			X	Head or neck
HP:0000707	*Abnormality of the nervous system*			X	Nervous system
HP:0000716	*Depression*			X	Nervous system
HP:0000739	*Anxiety*			X	Nervous system
HP:0000750	*Delayed speech and language development*	X			Nervous system
HP:0000772	*Abnormal rib morphology*			X	Musculoskeletal system
HP:0000789	*Infertility*			X	Genitourinary system
HP:0000798	*Oligozoospermia*			X	Genitourinary system
HP:0000813	*Bicornuate uterus*		X	X	Genitourinary system
HP:0000815	*Hypergonadotropic hypogonadism*	X			Endocrine system
HP:0000819	*Diabetes mellitus*			X	Metabolism/homeostasis
HP:0000821	*Hypothyroidism*	X		X	Endocrine system
HP:0000823	*Delayed puberty*			X	Endocrine system
HP:0000824	*Decreased response to growth hormone stimulation test*	X			Endocrine system
HP:0000855	*Insulin resistance*			X	Metabolism/homeostasis
HP:0000858	*Irregular menstruation*			X	Genitourinary system
HP:0000864	*Abnormality of the hypothalamus-pituitary axis*		X		Endocrine system
HP:0000868	*Decreased fertility in females*			X	Genitourinary system
HP:0000876	*Oligomenorrhea*			X	Genitourinary system
HP:0000902	*Rib fusion*	X			Musculoskeletal system
HP:0000912	*Sprengel anomaly*			X	Musculoskeletal system
HP:0000924	*Abnormality of the skeletal system*			X	Musculoskeletal system
HP:0000939	*Osteoporosis*			X	Musculoskeletal system

HP:0000953	*Hyperpigmentation of the skin*	X		X	Integument
HP:0000957	*Cafe-au-lait spot*	X		X	Integument
HP:0000958	*Dry skin*			X	Integument
HP:0000960	*Sacral dimple*	X			Integument
HP:0000967	*Petechiae*			X	Integument
HP:0000978	*Bruising susceptibility*	X		X	Blood and blood-forming tissues
HP:0000979	*Purpura*			X	Integument
HP:0001000	*Abnormality of skin pigmentation*	X	X	X	Integument
HP:0001010	*Hypopigmentation of the skin*			X	Integument
HP:0001017	*Anemic pallor*	X			Integument
HP:0001045	*Vitiligo*	X			Integument
HP:0001053	*Hypopigmented skin patches*		X		Integument
HP:0001072	*Thickened skin*			X	Integument
HP:0001097	*Keratoconjunctivitis sicca*			X	Eye
HP:0001155	*Abnormality of the hand*			X	Musculoskeletal system
HP:0001156	*Brachydactyly*			X	Musculoskeletal system
HP:0001159	*Syndactyly*	X			Musculoskeletal system
HP:0001166	*Arachnodactyly*			X	Musculoskeletal system
HP:0001172	*Abnormal thumb morphology*	X	X	X	Musculoskeletal system
HP:0001177	*Preaxial hand polydactyly*	X		X	Musculoskeletal system
HP:0001195	*Single umbilical artery*	X			Prenatal development or birth
HP:0001199	*Triphalangeal thumb*		X	X	Musculoskeletal system
HP:0001233	*2-3 finger cutaneous syndactyly*	X			Musculoskeletal system
HP:0001238	*Slender finger*			X	Musculoskeletal system
HP:0001245	*Small thenar eminence*	X		X	musculature
HP:0001249	*Intellectual disability*	X	X		Nervous system
HP:0001251	*Ataxia*	X			Nervous system
HP:0001252	*Hypotonia*	X			musculature
HP:0001263	*Global developmental delay*	X	X		Nervous system
HP:0001273	*Abnormal corpus callosum morphology*			X	Nervous system
HP:0001274	*Agenesis of corpus callosum*	X			Nervous system
HP:0001321	*Cerebellar hypoplasia*	X			Nervous system
HP:0001328	*Specific learning disability*	X			Nervous system
HP:0001331	*Absent septum pellucidum*	X		X	Nervous system
HP:0001347	*Hyperreflexia*		X	X	Nervous system
HP:0001360	*Holoprosencephaly*			X	Nervous system
HP:0001363	*Craniosynostosis*			X	Musculoskeletal system
HP:0001371	*Flexion contracture*	X			Connective tissue
HP:0001374	*Congenital hip dislocation*			X	Musculoskeletal system
HP:0001385	*Hip dysplasia*			X	Musculoskeletal system
HP:0001392	*Abnormality of the liver*		X		Digestive system
HP:0001498	*Carpal bone hypoplasia*			X	Musculoskeletal system
HP:0001508	*Failure to thrive*	X		X	Growth abnormality

HP:0001510	*Growth delay*	X	X	X	Growth abnormality
HP:0001511	*Intrauterine growth retardation*	X	X	X	Growth abnormality
HP:0001513	*Obesity*			X	Growth abnormality
HP:0001518	*Small for gestational age*	X		X	Growth abnormality
HP:0001537	*Umbilical hernia*		X		Digestive system
HP:0001545	*Anteriorly placed anus*	X			Digestive system
HP:0001561	*Polyhydramnios*	X			Prenatal development or birth
HP:0001562	*Oligohydramnios*		X		Prenatal development or birth
HP:0001572	*Macrodontia*	X			Musculoskeletal system
HP:0001627	*Abnormal heart morphology*	X			Cardiovascular system
HP:0001629	*Ventricular septal defect*	X		X	Cardiovascular system
HP:0001631	*Atrial septal defect*	X	X	X	Cardiovascular system
HP:0001636	*Tetralogy of Fallot*	X	X	X	Cardiovascular system
HP:0001638	*Cardiomyopathy*			X	musculature
HP:0001639	*Hypertrophic cardiomyopathy*		X		Cardiovascular system
HP:0001642	*Pulmonic stenosis*			X	Cardiovascular system
HP:0001643	*Patent ductus arteriosus*	X	X	X	Cardiovascular system
HP:0001646	*Abnormal aortic valve morphology*		X		Cardiovascular system
HP:0001650	*Aortic valve stenosis*			X	Cardiovascular system
HP:0001651	*Dextrocardia*	X			Cardiovascular system
HP:0001655	*Patent foramen ovale*	X			Cardiovascular system
HP:0001662	*Bradycardia*	X			Cardiovascular system
HP:0001671	*Abnormal cardiac septum morphology*		X		Cardiovascular system
HP:0001674	*Complete atrioventricular canal defect*	X			Cardiovascular system
HP:0001679	*Abnormal aortic morphology*		X		Cardiovascular system
HP:0001680	*Coarctation of aorta*	X		X	Cardiovascular system
HP:0001734	*Annular pancreas*	X		X	Endocrine system
HP:0001741	*Phimosis*			X	Genitourinary system
HP:0001748	*Polysplenia*	X			Immune system
HP:0001760	*Abnormal foot morphology*		X		Musculoskeletal system
HP:0001762	*Talipes equinovarus*	X		X	Connective tissue
HP:0001763	*Pes planus*		X		Musculoskeletal system
HP:0001770	*Toe syndactyly*		X		Musculoskeletal system
HP:0001776	*Bilateral talipes equinovarus*	X			Connective tissue
HP:0001824	*Weight loss*		X		Growth abnormality
HP:0001864	*Clinodactyly of the 5th toe*	X			Musculoskeletal system
HP:0001871	*Abnormality of blood and blood-forming tissues*		X		Blood and blood-forming tissues
HP:0001873	*Thrombocytopenia*	X	X	X	Blood and blood-forming tissues
HP:0001875	*Decreased total neutrophil count*	X		X	Immune system
HP:0001876	*Pancytopenia*	X			Blood and blood-forming tissues
HP:0001882	*Decreased total leukocyte count*	X	X		Immune system

HP:0001896	*Reticulocytopenia*	X			Blood and blood-forming tissues
HP:0001903	*Anemia*	X	X	X	Blood and blood-forming tissues
HP:0001909	*Leukemia*	X		X	Neoplasm
HP:0001915	*Aplastic anemia*	X		X	Blood and blood-forming tissues
HP:0001963	*Abnormal speech discrimination*			X	Ear
HP:0002007	*Frontal bossing*		X	X	Head or neck
HP:0002014	*Diarrhea*			X	Digestive system
HP:0002015	*Dysphagia*			X	Digestive system
HP:0002018	*Nausea*			X	Digestive system
HP:0002019	*Constipation*			X	Digestive system
HP:0002020	*Gastroesophageal reflux*			X	Digestive system
HP:0002023	*Anal atresia*	X	X	X	Digestive system
HP:0002024	*Malabsorption*			X	Digestive system
HP:0002027	*Abdominal pain*			X	Digestive system
HP:0002032	*Esophageal atresia*	X		X	Digestive system
HP:0002079	*Hypoplasia of the corpus callosum*	X			Nervous system
HP:0002089	*Pulmonary hypoplasia*	X			Respiratory system
HP:0002090	*Pneumonia*	X			Respiratory system
HP:0002101	*Abnormal lung lobation*	X			Respiratory system
HP:0002119	*Ventriculomegaly*	X	X	X	Nervous system
HP:0002126	*Polymicrogyria*	X			Nervous system
HP:0002144	*Tethered cord*	X			Nervous system
HP:0002188	*Delayed CNS myelination*	X			Nervous system
HP:0002206	*Pulmonary fibrosis*			X	Respiratory system
HP:0002245	*Meckel diverticulum*		X		Digestive system
HP:0002247	*Duodenal atresia*	X		X	Digestive system
HP:0002251	*Aganglionic megacolon*		X		Nervous system
HP:0002308	*Chiari malformation*	X		X	Nervous system
HP:0002414	*Spina bifida*		X	X	Musculoskeletal system
HP:0002518	*Abnormal periventricular white matter morphology*	X			Nervous system
HP:0002566	*Intestinal malrotation*			X	Digestive system
HP:0002575	*Tracheoesophageal fistula*	X	X	X	Digestive system
HP:0002578	*Gastroparesis*			X	musculature
HP:0002607	*Bowel incontinence*			X	Digestive system
HP:0002650	*Scoliosis*	X	X	X	Musculoskeletal system
HP:0002664	*Neoplasm*		X	X	Neoplasm
HP:0002667	*Nephroblastoma*	X		X	Neoplasm
HP:0002671	*Basal cell carcinoma*			X	Neoplasm
HP:0002691	*Platybasia*			X	Musculoskeletal system
HP:0002718	*Recurrent bacterial infections*			X	Immune system
HP:0002719	*Recurrent infections*			X	Immune system
HP:0002808	*Kyphosis*			X	Musculoskeletal system

HP:0002814	*Abnormality of the lower limb*			X	Musculoskeletal system
HP:0002817	*Abnormality of the upper limb*		X	X	Musculoskeletal system
HP:0002823	*Abnormal femur morphology*		X		Musculoskeletal system
HP:0002827	*Hip dislocation*		X		Musculoskeletal system
HP:0002841	*Recurrent fungal infections*			X	Immune system
HP:0002860	*Squamous cell carcinoma*	X		X	Neoplasm
HP:0002861	*Melanoma*			X	Neoplasm
HP:0002863	*Myelodysplasia*	X	X	X	Neoplasm
HP:0002885	*Medulloblastoma*	X		X	Neoplasm
HP:0002949	*Fused cervical vertebrae*	X			Musculoskeletal system
HP:0002984	*Hypoplasia of the radius*	X		X	Musculoskeletal system
HP:0002996	*Limited elbow movement*			X	Musculoskeletal system
HP:0003002	*Breast carcinoma*	X			Neoplasm
HP:0003006	*Neuroblastoma*	X		X	Neoplasm
HP:0003022	*Hypoplasia of the ulna*		X		Musculoskeletal system
HP:0003031	*Ulnar bowing*			X	Musculoskeletal system
HP:0003074	*Hyperglycemia*			X	Metabolism/homeostasis
HP:0003119	*Abnormal circulating lipid concentration*			X	Metabolism/homeostasis
HP:0003213	*Deficient excision of UV-induced pyrimidine dimers in DNA*	X			Metabolism/homeostasis
HP:0003214	*Prolonged G2 phase of cell cycle*	X			Metabolism/homeostasis
HP:0003220	*Abnormality of chromosome stability*	X	X		Metabolism/homeostasis
HP:0003221	*Chromosomal breakage induced by crosslinking agents*	X		X	Metabolism/homeostasis
HP:0003241	*External genital hypoplasia*	X		X	Genitourinary system
HP:0003250	*Aplasia of the vagina*			X	Genitourinary system
HP:0003251	*Male infertility*	X			Genitourinary system
HP:0003254	*Abnormality of DNA repair*			X	Metabolism/homeostasis
HP:0003272	*Abnormal hip bone morphology*			X	Musculoskeletal system
HP:0003452	*Increased circulating iron concentration*			X	Metabolism/homeostasis
HP:0003468	*Abnormal vertebral morphology*	X		X	Musculoskeletal system
HP:0003764	*Nevus*	X			Integument
HP:0003774	*Stage 5 chronic kidney disease*	X			Genitourinary system
HP:0003834	*Shoulder dislocation*	X			Musculoskeletal system
HP:0003956	*Bowed forearm bones*			X	Musculoskeletal system
HP:0003974	*Absent radius*	X		X	Musculoskeletal system
HP:0003982	*Aplasia of the ulna*			X	Musculoskeletal system
HP:0004209	*Clinodactyly of the 5th finger*	X	X		Musculoskeletal system
HP:0004247	*Small scaphoid*			X	Musculoskeletal system
HP:0004253	*Absent trapezium*			X	Musculoskeletal system
HP:0004255	*Small trapezium*			X	Musculoskeletal system
HP:0004322	*Short stature*	X	X	X	Growth abnormality

HP:0004323	*Abnormality of body weight*			X	Growth abnormality
HP:0004325	*Decreased body weight*	X			Growth abnormality
HP:0004349	*Reduced bone mineral density*		X	X	Musculoskeletal system
HP:0004395	*Malnutrition*			X	Digestive system
HP:0004429	*Recurrent viral infections*			X	Immune system
HP:0004510	*Pancreatic islet-cell hyperplasia*	X			Endocrine system
HP:0004590	*Hypoplastic sacrum*	X		X	Musculoskeletal system
HP:0004602	*Cervical C2/C3 vertebral fusion*			X	Musculoskeletal system
HP:0004712	*Renal malrotation*			X	Genitourinary system
HP:0004808	*Acute myeloid leukemia*	X		X	Neoplasm
HP:0004935	*Pulmonary artery atresia*			X	Cardiovascular system
HP:0004977	*Bilateral radial aplasia*	X			Musculoskeletal system
HP:0005214	*Intestinal obstruction*			X	Digestive system
HP:0005343	*Hypoplasia of the bladder*	X			Genitourinary system
HP:0005344	*Abnormal carotid artery morphology*		X		Cardiovascular system
HP:0005473	*Fusion of middle ear ossicles*			X	Musculoskeletal system
HP:0005518	*Increased mean corpuscular volume*	X			Blood and blood-forming tissues
HP:0005522	*Pyridoxine-responsive sideroblastic anemia*		X		Blood and blood-forming tissues
HP:0005528	*Bone marrow hypocellularity*	X		X	Blood and blood-forming tissues
HP:0005632	*Absent forearm*	X			Musculoskeletal system
HP:0005709	*2-3 toe cutaneous syndactyly*	X			Musculoskeletal system
HP:0005743	*Avascular necrosis of the capital femoral epiphysis*			X	Musculoskeletal system
HP:0005792	*Short humerus*	X		X	Musculoskeletal system
HP:0005912	*Biliary atresia*	X		X	Digestive system
HP:0006101	*Finger syndactyly*		X		Musculoskeletal system
HP:0006190	*Radially deviated wrists*			X	Musculoskeletal system
HP:0006248	*Limited wrist movement*			X	Musculoskeletal system
HP:0006254	*Elevated circulating alpha-fetoprotein concentration*	X			Metabolism/homeostasis
HP:0006265	*Aplasia/Hypoplasia of fingers*		X		Musculoskeletal system
HP:0006349	*Agenesis of permanent teeth*	X			Musculoskeletal system
HP:0006433	*Radial ray deficiency*	X		X	Musculoskeletal system
HP:0006482	*Abnormal dental morphology*			X	Musculoskeletal system
HP:0006501	*Aplasia/Hypoplasia of the radius*		X	X	Musculoskeletal system
HP:0006660	*Aplastic clavicle*			X	Musculoskeletal system
HP:0006727	*T-cell acute lymphoblastic leukemias*	X			Neoplasm
HP:0006824	*Cranial nerve paralysis*		X		Nervous system
HP:0007018	*Attention deficit hyperactivity disorder*	X			Nervous system
HP:0007099	*Chiari type I malformation*	X			Nervous system
HP:0007400	*Irregular hyperpigmentation*		X		Integument

HP:0007565	*Multiple cafe-au-lait spots*	X	X		Integument
HP:0007587	*Numerous pigmented freckles*			X	Integument
HP:0007766	*Optic disc hypoplasia*	X			Eye
HP:0007874	*Almond-shaped palpebral fissure*		X	X	Head or neck
HP:0008053	*Aplasia/Hypoplasia of the iris*		X		Eye
HP:0008070	*Sparse hair*	X			Integument
HP:0008209	*Premature ovarian insufficiency*			X	Genitourinary system
HP:0008551	*Microtia*	X		X	Ear
HP:0008661	*Urethral stenosis*			X	Genitourinary system
HP:0008678	*Renal hypoplasia/aplasia*		X		Genitourinary system
HP:0008734	*Decreased testicular size*			X	Genitourinary system
HP:0008839	*Hypoplastic pelvis*			X	Musculoskeletal system
HP:0008897	*Postnatal growth retardation*	X			Growth abnormality
HP:0009592	*Astrocytoma*			X	Neoplasm
HP:0009603	*Deviation of the thumb*			X	Musculoskeletal system
HP:0009623	*Proximal placement of thumb*	X			Musculoskeletal system
HP:0009660	*Short phalanx of the thumb*			X	Musculoskeletal system
HP:0009777	*Absent thumb*	X		X	Musculoskeletal system
HP:0009778	*Short thumb*	X		X	Musculoskeletal system
HP:0009804	*Tooth agenesis*			X	Musculoskeletal system
HP:0009821	*Forearm undergrowth*			X	Musculoskeletal system
HP:0009829	*Phocomelia*	X			Musculoskeletal system
HP:0009892	*Anotia*	X		X	Ear
HP:0009942	*Duplication of thumb phalanx*	X		X	Musculoskeletal system
HP:0009943	*Complete duplication of thumb phalanx*	X			Musculoskeletal system
HP:0009944	*Partial duplication of thumb phalanx*	X		X	Musculoskeletal system
HP:0010034	*Short 1st metacarpal*	X			Musculoskeletal system
HP:0010035	*Aplasia of the 1st metacarpal*	X			Musculoskeletal system
HP:0010293	*Aplasia/Hypoplasia of the uvula*		X		Head or neck
HP:0010305	*Absence of the sacrum*			X	Musculoskeletal system
HP:0010442	*Polydactyly*			X	Musculoskeletal system
HP:0010445	*Primum atrial septal defect*	X			Cardiovascular system
HP:0010461	*Abnormality of the male genitalia*			X	Genitourinary system
HP:0010469	*Absent testis*		X	X	Genitourinary system
HP:0010628	*Facial palsy*	X		X	Nervous system
HP:0010664	*Fusion of the left and right thalami*	X			Nervous system
HP:0010704	*1-2 finger cutaneous syndactyly*			X	Musculoskeletal system
HP:0011014	*Abnormal glucose homeostasis*			X	Metabolism/homeostasis
HP:0011069	*Supernumerary tooth*			X	Musculoskeletal system
HP:0011107	*Recurrent aphthous stomatitis*			X	Head or neck
HP:0011109	*Chronic sinusitis*			X	Respiratory system

HP:0011133	*Increased sensitivity to ionizing radiation*			X	Metabolism/homeostasis
HP:0011419	*Placental abruption*	X			Prenatal development or birth
HP:0011590	*Double aortic arch*			X	Cardiovascular system
HP:0011800	*Midface retrusion*	X		X	Head or neck
HP:0011834	*Moyamoya phenomenon*			X	Nervous system
HP:0011835	*Absent scaphoid*	X		X	Musculoskeletal system
HP:0011940	*Anterior wedging of T12*	X			Musculoskeletal system
HP:0011968	*Feeding difficulties*	X		X	Digestive system
HP:0012041	*Decreased fertility in males*		X		Genitourinary system
HP:0012165	*Oligodactyly*	X			Musculoskeletal system
HP:0012174	*Glioblastoma multiforme*			X	Neoplasm
HP:0012182	*Oropharyngeal squamous cell carcinoma*			X	Neoplasm
HP:0012210	*Abnormal renal morphology*	X	X	X	Genitourinary system
HP:0012285	*Abnormal hypothalamus physiology*			X	Endocrine system
HP:0012506	*Small pituitary gland*	X			Endocrine system
HP:0012622	*Chronic kidney disease*			X	Genitourinary system
HP:0012639	*Abnormal nervous system morphology*		X		Nervous system
HP:0012745	*Short palpebral fissure*	X	X	X	Head or neck
HP:0012799	*Unilateral facial palsy*	X			Nervous system
HP:0020073	*Hypopigmented macule*	X			Integument
HP:0020128	*Aplasia of the olfactory tract*	X			Nervous system
HP:0025023	*Rectal atresia*	X			Digestive system
HP:0025031	*Abnormality of the digestive system*			X	Digestive system
HP:0025127	*Actinic keratosis*			X	Integument
HP:0025261	*Stiff finger*			X	Musculoskeletal system
HP:0025318	*Ovarian carcinoma*	X			Neoplasm
HP:0025474	*Erythematous plaque*			X	Integument
HP:0025502	*Overweight*			X	Growth abnormality
HP:0030048	*Colpocephaly*	X			Nervous system
HP:0030079	*Cervix cancer*			X	Neoplasm
HP:0030084	*Clinodactyly*	X		X	Musculoskeletal system
HP:0030260	*Microphallus*	X		X	Genitourinary system
HP:0030283	*Partial absence of the septum pellucidum*	X			Nervous system
HP:0030680	*Abnormal cardiovascular system morphology*	X			Cardiovascular system
HP:0030996	*Megaduodenum*			X	Digestive system
HP:0031095	*Abnormal humerus morphology*			X	Musculoskeletal system
HP:0031640	*Abnormal radial artery morphology*			X	Cardiovascular system
HP:0031689	*Megakaryocyte dysplasia*	X			Blood and blood-forming tissues
HP:0031703	*Abnormal ear morphology*			X	Ear
HP:0031936	*Delayed ability to walk*	X			Nervous system

HP:0031965	*Increased RBC distribution width*	X			Blood and blood-forming tissues
HP:0032043	*Odynophagia*			X	Digestive system
HP:0032154	*Aphthous ulcer*			X	Head or neck
HP:0032188	*Cellular hypersensitivity to mitomycin C*	X			Metabolism/homeostasis
HP:0032464	*Ureteral hypoplasia*	X			Genitourinary system
HP:0033183	*Bilobed right lung*	X			Respiratory system
HP:0033667	*Diminished mental health*			X	Other/unclassified
HP:0033725	*Thin corpus callosum*	X			Nervous system
HP:0034057	*Fetal anomaly*			X	Prenatal development or birth
HP:0034231	*Sigmoid kidney*			X	Genitourinary system
HP:0034323	*Reduced circulating growth hormone concentration*	X		X	Endocrine system
HP:0034585	*Cochlear nerve hypoplasia*			X	Nervous system
HP:0034681	*Finger joint contracture*			X	Connective tissue
HP:0034976	*Absent pituitary stalk*	X			Endocrine system
HP:0040012	*Chromosome breakage*	X			Metabolism/homeostasis
HP:0040071	*Abnormal morphology of ulna*		X		Musculoskeletal system
HP:0040075	*Hypopituitarism*	X			Endocrine system
HP:0040090	*Abnormal tympanic membrane morphology*			X	Ear
HP:0040183	*Encopresis*			X	Digestive system
HP:0040189	*Scaling skin*			X	Integument
HP:0040270	*Impaired glucose tolerance*			X	Metabolism/homeostasis
HP:0045005	*Neural tube defect*			X	Nervous system
HP:0045025	*Narrow palpebral fissure*			X	Head or neck
HP:0100026	*Arteriovenous malformation*		X		Cardiovascular system
HP:0100542	*Abnormal localization of kidney*		X		Genitourinary system
HP:0100559	*Lower limb asymmetry*			X	Growth abnormality
HP:0100587	*Abnormal preputium morphology*		X		Genitourinary system
HP:0100615	*Ovarian Neoplasm*	X			Neoplasm
HP:0100760	*Clubbing of toes*		X		Musculoskeletal system
HP:0100842	*Septo-optic dysplasia*	X			Nervous system
HP:0100867	*Duodenal stenosis*		X		Digestive system
HP:0200036	*Skin nodule*			X	Integument
HP:0200043	*Verrucae*			X	Neoplasm
HP:0410028	*Recurrent oral herpes*			X	Immune system
HP:5200320	*Diminishment of relationship seeking*			X	Nervous system
HP:6000064	*Excessive eructation*			X	Digestive system
HP:6000942	*Thumb hypoplasia grade 4*			X	Musculoskeletal system
